# Multimodal Hippocampal Subfield Grading For Alzheimer’s Disease Classification

**DOI:** 10.1038/s41598-019-49970-9

**Published:** 2019-09-25

**Authors:** Kilian Hett, Vinh-Thong Ta, Gwenaëlle Catheline, Thomas Tourdias, José V. Manjón, Pierrick Coupé, Alzheimer’s Disease Neuroimaging Initiative, Michael W. Weiner, Paul Aisen, Ronald Petersen, Clifford R. Jack, William Jagust, John Q. Trojanowki, Arthur W. Toga, Laurel Beckett, Robert C. Green, Andrew J. Saykin, John Morris, Leslie M. Shaw, Zaven Khachaturian, Greg Sorensen, Maria Carrillo, Lew Kuller, Marc Raichle, Steven Paul, Peter Davies, Howard Fillit, Franz Hefti, Davie Holtzman, M. Marcel Mesulam, William Potter, Peter Snyder, Tom Montine, Ronald G. Thomas, Michael Donohue, Sarah Walter, Tamie Sather, Gus Jiminez, Archana B. Balasubramanian, Jennifer Mason, Iris Sim, Danielle Harvey, Matthew Bernstein, Nick Fox, Paul Thompson, Norbert Schuff, Charles DeCArli, Bret Borowski, Jeff Gunter, Matt Senjem, Prashanthi Vemuri, David Jones, Kejal Kantarci, Chad Ward, Robert A. Koeppe, Norm Foster, Eric M. Reiman, Kewei Chen, Chet Mathis, Susan Landau, Nigel J. Cairns, Erin Householder, Lisa Taylor-Reinwald, Virginia Lee, Magdalena Korecka, Michal Figurski, Karen Crawford, Scott Neu, Tatiana M. Foroud, Steven Potkin, Li Shen, Kelley Faber, Sungeun Kim, Kwangsik Nho, Lean Thal, Richard Frank, John Hsiao, Jeffrey Kaye, Joseph Quinn, Lisa Silbert, Betty Lind, Raina Carter, Sara Dolen, Beau Ances, Maria Carroll, Mary L. Creech, Erin Franklin, Mark A. Mintun, Stacy Schneider, Angela Oliver, Lon S. Schneider, Sonia Pawluczyk, Mauricio Beccera, Liberty Teodoro, Bryan M. Spann, James Brewer, Helen Vanderswag, Adam Fleisher, Daniel Marson, Randall Griffith, David Clark, David Geldmacher, John Brockington, Erik Roberson, Marissa Natelson Love, Judith L. Heidebrink, Joanne L. Lord, Sara S. Mason, Colleen S. Albers, David Knopman, Kris Johnson, Hillel Grossman, Effie Mitsis, Raj C. Shah, Leyla deToledo-Morrell, Rachelle S. Doody, Javier Villanueva-Meyer, Munir Chowdhury, Susan Rountree, Mimi Dang, Ranjan Duara, Daniel Varon, Maria T. Greig, Peggy Roberts, Yaakov Stern, Lawrence S. Honig, Karen L. Bell, Marilyn Albert, Chiadi Onyike, Daniel D’Agostino, Stephanie Kielb, James E. Galvin, Brittany Cerbone, Christina A. Michel, Dana M. Pogorelec, Henry Rusinek, Mony J de Leon, Lidia Glodzik, Susan De Santi, Kyle Womack, Dana Mathews, Mary Quiceno, P. Murali Doraiswamy, Jeffrey R. Petrella, Salvador Borges-Neto, Terence Z. Wong, Edward Coleman, Allan I. Levey, James J. Lah, Janet S. Cella, Jeffrey M. Burns, Russell H. Swerdlow, William M. Brooks, Steven E. Arnold, Jason H. Karlawish, David Wolk, Christopher M. Clark, Liana Apostolova, Kathleen Tingus, Ellen Woo, Daniel H. S. Silverman, Po H. Lu, George Bartzokis, Charles D. Smith, Greg Jicha, Peter Hardy, Partha Sinha, Elizabeth Oates, Gary Conrad, Neill R Graff-Radford, Francine Parfitt, Tracy Kendall, Heather Johnson, Oscar L. Lopez, MaryAnn Oakley, Donna M. Simpson, Martin R. Farlow, Ann Marie Hake, Brandy R. Matthews, Jared R. Brosch, Scott Herring, Cynthia Hunt, Anton P. Porsteinsson, Bonnie S. Goldstein, Kim Martin, Kelly M. Makino, M. Saleem Ismail, Connie Brand, Ruth A. Mulnard, Gaby Thai, Catherine Mc-Adams-Ortiz, Christopher H. van Dyck, Richard E. Carson, Martha G. MacAvoy, Pradeep Varma, Howard Chertkow, Howard Bergman, Chris Hosein, Sandra Black, Bojana Stefanovic, Curtis Caldwell, Ging-Yuek Robin Hsiung, Howard Feldman, Benita Mudge, Michele Assaly, Elizabeth Finger, Stephen Pasternack, Irina Rachisky, Dick Trost, Andrew Kertesz, Charles Bernick, Donna Munic, Kristine Lipowski, M. A. Sandra Weintraub, Borna Bonakdarpour, Diana Kerwin, Chuang-Kuo Wu, Nancy Johnson, Carl Sadowsky, Teresa Villena, Raymond Scott Turner, Kathleen Johnson, Brigid Reynolds, Reisa A. Sperling, Keith A. Johnson, Gad Marshall, Jerome Yesavage, Joy L. Taylor, Barton Lane, Allyson Rosen, Jared Tinklenberg, Marwan N. Sabbagh, Christine M. Belden, Sandra A. Jacobson, Sherye A. Sirrel, Neil Kowall, Ronald Killiany, Andrew E. Budson, Alexander Norbash, Patricia Lynn Johnson, Thomas O. Obisesan, Saba Wolday, Joanne Allard, Alan Lerner, Paula Ogrocki, Curtis Tatsuoka, Parianne Fatica, Evan Fletcher, Pauline Maillard, John Olichney, Owen Carmichael, Smita Kittur, Michael Borrie, T-Y Lee, Rob Bartha, Sterling Johnson, Sanjay Asthana, Cynthia M. Carlsson, Adrian Preda, Dana Nguyen, Pierre Tariot, Anna Burke, Nadira Trncic, Adam Fleisher, Stephanie Reeder, Vernice Bates, Horacio Capote, Michelle Rainka, Douglas W. Scharre, Maria Kataki, Anahita Adeli, Earl A. Zimmerman, Dzintra Celmins, Alice D. Brown, Godfrey D. Pearlson, Karen Blank, Karen Anderson, Laura A. Flashman, Marc Seltzer, Mary L. Hynes, Robert B. Santulli, Kaycee M. Sink, Leslie Gordineer, Jeff D. Williamson, Pradeep Garg, Franklin Watkins, Brian R. Ott, Henry Querfurth, Geoffrey Tremont, Stephen Salloway, Paul Malloy, Stephen Correia, Howard J. Rosen, Bruce L. Miller, David Perry, Jacobo Mintzer, Kenneth Spicer, David Bachman, Elizabether Finger, Stephen Pasternak, Irina Rachinsky, John Rogers, Dick Drost, Nunzio Pomara, Raymundo Hernando, Antero Sarrael, Susan K. Schultz, Laura L. Boles Ponto, Hyungsub Shim, Karen Ekstam Smith, Norman Relkin, Gloria Chaing, Michael Lin, Lisa Ravdin, Amanda Smith, Balebail Ashok Raj, Kristin Fargher

**Affiliations:** 1 Univ. Bordeaux, LaBRI, UMR 5800, PICTURA, F-33400 Talence, France; 2 Bordeaux INP, LaBRI, UMR 5800, PICTURA, F-33405 Talence, France; 3 CNRS, LaBRI, UMR 5800, PICTURA, F-33400 Talence, France; 4 Univ. Bordeaux, INCIA, UMR 5287, F-33400 Talence, France; 5 CNRS, INCIA, UMR 5287, F-33400 Talence, France; 6 CHU de Bordeaux, Service de neuroimagerie diagnostique et thérapeutique, F-33076 Bordeaux, France; 7 Neurocentre Magendie, INSERM U1215, F-33077 Bordeaux, France; 8 Universitat Politècnia de València, ITACA, 46022 Valencia, Spain; 9 Univ. Bordeaux, F-33000 Bordeaux, France; 10 UC San Francisco, San Francisco, CA 94107 USA; 11 UC San Diego, La Jolla, CA 92093 USA; 12 Mayo Clinic, Rochester, MN USA; 13 UC Berkeley, Berkeley, San Francisco USA; 14 University of Pennsylvania, Philadelphia, PA 19104 USA; 15 USC, Los Angeles, CA 90032 USA; 16 UC Davis, Sacramento, CA USA; 17 Brigham and Women’s Hospital/Harvard Medical School, Boston, MA 02215 USA; 18 Indiana University, Bloomington, IN 47405 USA; 19 Washington University, St. Louis, MO 63110 USA; 20 Prevent Alzheimer’s Disease 2020, Rockville, MD 20850 USA; 21 Siemens, Erlangen, Germany; 22 Alzheimer’s Association, Chicago, IL 60631 USA; 23 University of Pittsburg, Pittsburgh, PA 15213 USA; 24 Cornell University, Ithaca, NY 14853 USA; 25 Albert Einstein College of Medicine of Yeshiva University, Bronx, NY 10461 USA; 26 AD Drug Discovery Foundation, New York, NY 10019 USA; 27 Acumen Pharmaceuticals, Livermore, CA 94551 USA; 28 Northwestern University, Chicago, IL 60611 USA; 29 National Institute of Mental Health, Bethesda, MD 20892 USA; 30 Brown University, Providence, RI 02912 USA; 31 University of Washington, Seattle, WA 98195 USA; 32 University of London, London, UK; 33 UCLA, Torrance, CA 90509 USA; 34 University of Michigan, Ann Arbor, MI 48109-2800 USA; 35 University of Utah, Salt Lake City, UT 84112 USA; 36 Banner Alzheimer’s Institute, Phoenix, AZ 85006 USA; 37 UUC Irvine, Orange, CA 92868 USA; 38 Johns Hopkins University, Baltimore, MD 21205 USA; 39 Richard Frank Consulting, New York, NY USA; 40 National Institute on Aging, Baltimore, Maryland USA; 41 Oregon Health and Science University, Portland, OR 97239 USA; 42 University of Alabama, Birmingham, AL USA; 43 Mount Sinai School of Medicine, New York, NY USA; 44 Rush University Medical Center, Chicago, IL 60612 USA; 45 Baylor College of Medicine, Houston, TX USA; 46 Wien Center, Miami Beach, FL 33140 USA; 47 Columbia University Medical Center, New York, NY USA; 48 New York University, New York, NY USA; 49 University of Texas Southwestern Medical School, Galveston, TX 77555 USA; 50 Duke University Medical Center, Durham, NC USA; 51 Emory University, Atlanta, GA 30307 USA; 52 University of Kansas Medical Center, Kansas City, Kansas USA; 53 University of Kentucky, Lexington, KY USA; 54 Mayo Clinic, Jacksonville, Florida USA; 55 University of Rochester Medical Center, Rochester, NY 14642 USA; 56 Yale University School of Medicine, New Haven, CT USA; 57 McGill Univ. Montreal-Jewish General Hospital, Montreal, PQ H3A 2A7 Canada; 58 Sunnybrook Health Sciences, Toronto, ON Canada; 59 U.B.C. Clinic for AD & Related Disorders, Vancouver, BC Canada; 60 Cognitive Neurology - St. Joseph’s, London, ON Canada; 61 Cleveland Clinic Lou Ruvo Center for Brain Health, Las Vegas, NV 89106 USA; 62 Premiere Research Inst (Palm Beach Neurology), W Palm Beach, FL USA; 63 Georgetown University Medical Center, Washington, DC 20007 USA; 64 Stanford University, Stanford, CA 94305 USA; 65 Boston University, Boston, Massachusetts USA; 66 Howard University, Washington, DC 20059 USA; 67 Case Western Reserve University, Cleveland, OH 44106 USA; 68 Neurological Care of CNY, Liverpool, NY 13088 USA; 69 St. Joseph’s Health Care, London, ON N6A 4H1 Canada; 70 Dent Neurologic Institute, Amherst, NY 14226 USA; 71 Ohio State University, Columbus, OH 43210 USA; 72 Albany Medical College, Albany, NY 12208 USA; 73 Hartford Hospital Olin Neuropsychiatry Research Center, Hartford, CT 06114 USA; 74 Dartmouth-Hitchcock Medical Center, Lebanon, NH USA; 75 Wake Forest University Health Sciences, Winston-Salem, NC USA; 76 Medical University South Carolina, Charleston, SC 29425 USA; 77 Nathan Kline Institute, Orangeburg, NY USA; 78 University of Iowa College of Medicine, Iowa City, IA 52242 USA; 79 University of South Florida: USF Health Byrd Alzheimer’s Institute, Tampa, FL 33613 USA

**Keywords:** Alzheimer's disease, Diagnostic markers, Predictive markers

## Abstract

Numerous studies have proposed biomarkers based on magnetic resonance imaging (MRI) to detect and predict the risk of evolution toward Alzheimer’s disease (AD). Most of these methods have focused on the hippocampus, which is known to be one of the earliest structures impacted by the disease. To date, patch-based grading approaches provide among the best biomarkers based on the hippocampus. However, this structure is complex and is divided into different subfields, not equally impacted by AD. Former *in*-*vivo* imaging studies mainly investigated structural alterations of these subfields using volumetric measurements and microstructural modifications with mean diffusivity measurements. The aim of our work is to improve the current classification performances based on the hippocampus with a new multimodal patch-based framework combining structural and diffusivity MRI. The combination of these two MRI modalities enables the capture of subtle structural and microstructural alterations. Moreover, we propose to study the efficiency of this new framework applied to the hippocampal subfields. To this end, we compare the classification accuracy provided by the different hippocampal subfields using volume, mean diffusivity, and our novel multimodal patch-based grading framework combining structural and diffusion MRI. The experiments conducted in this work show that our new multimodal patch-based method applied to the whole hippocampus provides the most discriminating biomarker for advanced AD detection while our new framework applied into subiculum obtains the best results for AD prediction, improving by two percentage points the accuracy compared to the whole hippocampus.

## Introduction

Alzheimer’s disease (AD) is an irreversible neurodegenerative process leading to mental dysfunctions. Subjects presenting mild cognitive impairment (MCI) have a higher risk of developing AD^1^. To study the preclinical phase of the disease, the Alzheimer’s disease neuroimaging initiative (ADNI) has been set up based on two MCI definitions: early MCI (eMCI) and late MCI (lMCI). Subjects with eMCI have milder cognitive impairment than those with lMCI, both suffering from amnesic MCI^2^. Such clinical symptoms are caused by changes like synaptic and neuronal losses that lead to structural and microstructural alterations. Neuroimaging studies performed on AD subjects reveal that when an AD diagnosis is made, alterations of brain structure are already advanced, emphasizing the need to study the early stages of the disease.

The improvement of medical imaging techniques such as magnetic resonance imaging (MRI) has enabled the development of efficient biomarkers capable of detecting alterations caused by AD^3^. Over the past years, many methods have been proposed to perform automatic detection of alterations associated with AD. First, studies proposed methods based on specific regions of interest (ROI) capturing alterations at an anatomical scale. Among structures impacted by AD, previous investigations have been focused on the hippocampus^4–6^, entorhinal cortex (EC)^7–9^, parahippocampal gyrus, amygdala^10^, or parietal lobe^11,12^. Alterations of these structures are usually estimated using volume^13,14^, shape^15,16^, or cortical thickness^17,18^ measurements. Beside ROI-based methods, whole brain analysis performed on structural MRI (s-MRI) has been proposed to detect areas impacted by AD at a voxel scale. These methods are usually based on voxel-based morphometry (VBM) or tensor-based morphometry (TBM) frameworks^19^. It is interesting to note that both VBM and ROI-based studies confirmed that the medial temporal lobe is a key area in detecting the first signs of AD^20–25^. These studies also showed that the hippocampus is one of the earliest regions altered by AD in the medial temporal lobe^26^. Moreover, the hippocampus volume is one of the criteria that can be used to confirm the diagnosis of AD in clinical routines^27^. Recently, advanced methods were proposed to capture subtler structural alterations of the hippocampus^9,28–30^. Those techniques demonstrated an increase in detection and prediction performances at different AD stages when compared to volume-based methods^30^. Among them, patch-based grading (PBG) methods demonstrated competitive results to detect the earliest stages of AD before a clinical diagnosis can be made^9,29,31^. The main idea of this approach is to capture inter-subject pattern similarities via non-local comparisons between two groups of subjects. Such methods have shown their ability to predict AD more than seven years before the conversion to dementia^32^ and might enable a differential diagnosis^33,34^.

Thus, the hippocampus has been one of the most studied structures to diagnose AD. However, this structure is not homogeneous, so it is usually subdivided into different subfields. Initial efforts to define the hippocampus subfields were mainly based on cell size, shape, and connectivity^35^. The terminology differs across segmentation protocols^36^, but the most recognized definition^37^ divides hippocampus into the subiculum, the cornu ammonis (CA1/2/3/4), and the dentrate gyrus (DG). The CA1 subfield represents the biggest area in the hippocampus. It is composed of different layers called the stratum radiatum (SR), the stratum lacunosum (SL), the stratum molecular (SM), and the stratum pyramidale (SP). Interestingly, studies have shown that hippocampal subfields could have different functional specializations. It has been suggested that CA3 and DG might be responsible for encoding early retrieval^38,39^ while CA1 is responsible for consolidation, late retrieval and recognition^40–42^. Furthermore, hippocampal subfields are not equally impacted by AD^43–49^. Indeed, several MRI studies demonstrated that subfields are impacted differently according to AD stages. Postmortem and *in vivo* imaging studies showed that the CA1SR-L-M are the subfields impacted with the greatest atrophy in advanced AD^45,46,48^. Recently, it has been shown that the subiculum is the earliest affected hippocampal region^49,50^.

These studies indicate that a subfield analysis of hippocampus alterations at a finer scale with an analysis of the subiculum could provide better tools for AD detection and prediction. The subiculum lies between CA1 and the entorhinal cortex in the medial temporal lobe. It shows a columnar organization (parasubiculum, presubiculum, postsubiculum, prosubiculum) combined with a laminar organization and is the main output of the hippocampus. Aside from those from CA1, several other extrinsic afferents terminate within the subiculum from the temporal lobe cortex (entorhinal cortex, perirhinal cortex, parahippocampal cortex, and amygdala). The anterior thalamic nuclei also project densely upon the subicular complex. In terms of efferent pathways, the subiculum projects to more extrinsic sites than any other hippocampal area. Notably, the subiculum shows dense extrinsic projections toward the anterior thalamic nuclei, the mammillary bodies, and the retrospinal cortex. Regarding its function, the subiculum is implicated in working memory. Several rodent behavioral studies also have shown that subiculum lesions impair spatial memory tasks with spatial working memory having a higher sensitivity than reference memory^51^.

Although structural MRI is a valuable imaging technique for measuring global structural modifications, such modality is not able to capture microstructural degradation. However, the microstructural modifications caused by AD are believed to occur before the atrophy measured by structural MRI. Therefore, diffusion MRI (d-MRI) appears as a potential candidate in detecting the earliest sign of AD. Several diffusion tensor imaging (DTI) studies proposed automatic methods for detecting modifications of diffusion parameters into the whole white matter volume using machine learning techniques^52–54^. Others studies showed modifications of diffusion parameters for AD patients into specific white matter structures such as the corpus callosum^55,56^, the fornix^57^, the cingulum^55^, and also in gray matter tissues such as the hippocampus^58^. More advanced d-MRI studies using brain connectivity and fiber tracking have been proposed to extract features describing axonal fiber alterations^57,59,60^. Finally, it has been shown that the hippocampal mean diffusivity (MD) is correlated to pathology progression and thus could be used as an efficient biomarker of AD^61^. Moreover, it was demonstrated that MD increases with the development of AD in the gray matter^62–64^. Therefore, in previous work, we showed that patch-based features applied to DTI demonstrated competitive performances to classify the early stages of AD^65^. Although some studies showed the superiority of MD over volumetric measurement to detect early sign of AD, this difference remains unclear^66,67^. However, several methods showed the possibility of using volumetric and MD measurements to capture early alterations caused by AD^68,69^. Recently, a study combining volumetric measurements and mean diffusivity of hippocampus subfields demonstrated that the CA1 and subiculum are the most impacted subfields in late AD stage^50^.

All these elements indicate that a multimodal method based on hippocampal subfields using an advanced image analysis framework could improve AD detection and prediction. Consequently, in this paper, we propose the study of hippocampal subfield efficiencies using s-MRI and d-MRI modalities for AD detection and prediction. To that purpose, we have developed a novel multimodal patch-based grading fusion scheme to better capture such structural and microstructural alterations. First, we compare the performance of our novel method with volume and MD within the whole hippocampus. Secondly, we demonstrate state-of-the-art performances compared to more advanced d-MRI based methods. Finally, we study the efficiency of each hippocampal subfields in improving AD detection and prediction using volume, MD, and our multimodal patch-based grading method. Our results show that while PBG based on s-MRI obtains the best performance for AD diagnosis, d-MRI obtains the best performance for AD prognosis. Our novel multimodal patch-based grading method based on these two modalities obtains the best scores for both AD detection and prediction. These results highlight that our multimodal patch-based grading provides more robust features than PBG based on only a single modality. Moreover, we demonstrate that the study of the hippocampus at a finer scale improves AD prediction. The experiments conducted with our new multimodal patch-based grading show that the whole hippocampus provides better results for AD detection, but the subiculum is the best area for AD prediction.

## Materials

### Dataset

Data used in this work was obtained from the Alzheimer’s Disease Neuroimaging Initiative (ADNI) dataset (http://adni.loni.ucla.edu). ADNI is a North American campaign launched in 2003 with the aim of providing MRI, positron emission tomography scans, clinical neurological measures, and other biomarkers. This dataset includes AD patients, MCI, and control normal (CN) subjects. The group of MCI is composed of subjects who have abnormal memory dysfunctions. In this work, we used data from the ADNI-2 campaign that proposes eMCI and lMCI stages. The eMCI and lMCI subgroups were obtained with the Wechsler Scale-Revised Logistical Memory I and II tests in accordance with the education levels of each subject. ADNI-2 provides T1-weighted (T1w) MRI, DTI scans for 54 CN, 79 eMCI, 39 lMCI, and 47 AD subjects. Only patients who have T1w and DTI images were selected in our work. Hence, in this work, we used 52 CN, 99 MCI composed of 65 eMCI, 34 lMCI, and 38 AD instead of the whole initial ADNI-2 dataset. All MRI data and clinical status were collected at the baseline. The list of subjects involved in our experiments is available (http://bit.ly/scirep_mpbg_dataset). Table 1 shows the distribution of the data for each group. The s-MRI and d-MRI scans used for all considered subjects in this study were acquired with the same protocol (https://adni.loni.usc.edu/wp-content/uploads/2010/05/ADNI2_GE_3T_22.0_T2.pdf). T1w MRI acquisition protocol was done with the 3D accelerated sagittal IR-SPGR, according to the ADNI protocol^70^. The d-MRI is composed of 46 separate angles, 5 T2-weighted images with no diffusion sensitization (b0 images) and 41 directions (b = 1000 s/mm^2^). The d-MRI protocol was chosen to optimize the signal-to-noise ratio in a fixed scan time^71^. The native resolution of s-MRI and d-MRI was set to 1 mm^3^ and 2 mm^3^, respectively.

**Table 1:** Description of the dataset used in this work.

	CN	eMCI	lMCI	AD	P value
Number of subjects	52	65	34	38	
Age (years)	72.6 ± 5.9	73.0 ± 7.7	73.5 ± 6.6	73.8 ± 8.7	p = 0.80^*a*^
Gender (female/male)	29/23	39/26	21/13	20/18	*χ*^2^ = 3.12, p = 0.37^*b*^
MMSE	28.9 ± 1.2	28.2 ± 1.5	27.3 ± 1.8	23.4 ± 1.7	p < 0.01^a^*
CDR-SB	0.0 ± 0.1	1.2 ± 0.6	1.7 ± 0.8	4.6 ± 1.4	p < 0.01^a^*
RAVLT	45.4 ± 9.7	36.5 ± 10.2	30.7 ± 8.9	22.6 ± 7.0	p < 0.01^a^*
FAQ	0.2 ± 0.9	2.3 ± 3.7	4.3 ± 4.8	14.6 ± 6.6	p < 0.01^a^*
ADAS11	5.2 ± 3.0	8.1 ± 3.6	12.5 ± 4.9	20.2 ± 7.6	p < 0.01^a^*
ADAS13	8.4 ± 4.4	13.3 ± 5.4	20.2 ± 6.7	30.0 ± 9.0	p < 0.01^a^*

Data are provided by ADNI. MMSE: Mini-Mental State Examination; CDR-SB: Clinical Dementia Rating-Sum of Boxes; RAVLT: Rey’s Auditory Verbal Learning Test; FAQ: Functional Activity Questionnaire; ADAS(11/13): Alzheimer’s Disease Assessment Scale.

*Significant at p < 0.05.

^*a*^Chi-square test (df = 3).

^*b*^Kruskal–Wallis test (df = 3).

### MRI processing

T1w images were processed using the volBrain system^72^ (http://volbrain.upv.es). This system is based on an advanced pipeline providing automatic segmentation of different brain structures from T1w MRI. The preprocessing is based on (a) a denoising step with an adaptive non-local mean filter^73^, (b) an affine registration in the MNI space^74^, (c) a correction of the image inhomogeneities^75^ and (d) an intensity normalization.

**Figure 1:**
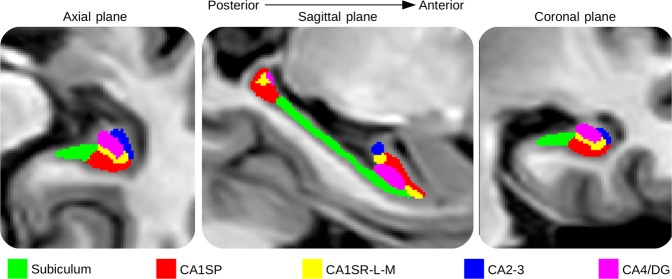
Segmentation of the hippocampal subfields. From left to right, segmentation maps of right hippocampal subfields displayed on the axial, sagittal and coronal plane.

Afterward, segmentation of hippocampal subfields was performed with HIPS^76^ based on a combination of non-linear registration and patch-based label fusion^77^. This method uses a training library based on a dataset composed of high-resolution T1w images manually labeled according to the protocol proposed by Winterburn *et al*.^37^. To perform the segmentation, the images are up-sampled with a local adaptive super-resolution method to fit the training image resolution^78^. The method provides automatic segmentation of hippocampal subfields gathered into five labels: Subiculum, CA1SP, CA1SR-L-M, CA2-3, and CA4/DG (see Fig. 1). Then, the segmentation maps obtained from the up-sampled T1w images were down-sampled to fit the MNI space resolution. All the following experiments were carried out with images into the MNI space. Finally, an estimation of the total intra-cranial volume was performed^79^.

### DTI processing

The preprocessing of the diffusion-weighted images is based on (a) a denoising step based on the LPCA filter^80^ and (b) a correction of the head motion using an affine registration. Afterward, we performed several steps to first obtain the mapping between the DWI native space and the MNI space and then to estimate the MD in the MNI space.

1. Estimation of the mapping between DWI native space and MNI space: First, a diffusion tensor model^81^ estimated at each voxel using Dipy library^82^. The resulting MD is first linearly registered to the CSF map obtained from the T1w in the MNI space. Then, the MD (in the MNI space) is non-linearly registered to the CSF map (in the MNI space) to compensate for echo-planar imaging (EPI) distortions^74^. Afterward, the affine transformation and the non-linear deformations are concatenated into a single transformation to obtain the final mapping (including EPI distortion correction) from the DWI native space to the MNI space. It must be noted that the MD map estimated in the DWI native space is only used to estimate the mapping between both spaces.
2. Estimation of the MD in the MNI space: The deformation field estimated at the previous step is used to register the *b*_0_ and each DWI direction from their native space into the MNI space using b-spline interpolations^74^. This is done to limit interpolation artifacts and to correct partial volume effect (PVE). It has been shown that up-sampling each DWI direction individually using interpolation before estimating DTI parameters enables the reduction of PVE present in DTI greatly^83^. Thus, the final diffusion tensor model is estimated in the MNI space using all the non-linearly registered DWI and *b*_0_.

To analyze microstructural modifications, the MD is estimated within each hippocampal subfield and the whole hippocampus structure with the segmentation described in the previous section. MD is defined as $\frac{{\lambda }_{1}+{\lambda }_{2}+{\lambda }_{3}}{3}$ where *λ*_1_, *λ*_2_, *λ*_3_ are the three eigenvalues of the fitted tensor.

Finally, quality control is conducted to exclude data presenting segmentation errors or misregistration after MRI and DTI preprocessing step. Thus, 10 CN subjects, 18 eMCI, 5 lMCI, and 9 AD patients have been excluded from the initial considered ADNI2 dataset (see the dataset used in our experiments Table 1).

## Methods

### Patch-based grading

Patch-based grading was first proposed for s-MRI^9^. The main idea of this exemplar-based method is to use the capability of patch-based techniques in order to capture subtle signal modifications related to anatomical degradations caused by AD. To date, the PBG methods demonstrate state-of-the-art performances in the detection of the earliest stage of AD^84^. To determine the pathological status of the subject under study, the PBG methods estimate the state of cerebral tissues at each voxel by a similarity measurement. This measurement is performed between the anatomical pattern of the subject under study and those extracted from two training populations, one healthy and another one unhealthy.

First, a training library *T* composed of two datasets of images is built: one with images from CN subjects and the other one from AD patients. Next, for each voxel *x*_*i*_ of the region of interest in the considered subject *x*, the PBG method produces a weak classifier denoted ${g}_{{x}_{i}}$. This weak classifier provides a surrogate of the pathological grading at the considered position. The weak classifier is computed using a measurement of the similarity between the patch ${P}_{{x}_{i}}$ surrounding the voxel *x*_*i*_ belonging to the image under study and a set ${K}_{{x}_{i}}$ of the closest patches extracted from the library *T*. The most similar patches are found using an approximative nearest neighbor method^85^. The grading value ${g}_{{x}_{i}}$ at *x*_*i*_ is defined as:

1 $${g}_{{x}_{i}}=\frac{{\sum }_{{t}_{j}\in {K}_{{x}_{i}}}\,w({P}_{{x}_{i}},{P}_{{t}_{j}}){p}_{t}}{{\sum }_{{t}_{j}\in {K}_{{x}_{i}}}\,w({P}_{{x}_{i}},{P}_{{t}_{j}})}$$

where ${P}_{{t}_{j}}$ is the patch surrounding the voxel *j* belonging to the training template $t\in T$, and $w({x}_{i},{t}_{j})$ is the weight assigned to the pathological status *p*_*t*_ of the training image *t*. We estimate *w* such that:

2 $$w({P}_{{x}_{i}},{P}_{{t}_{j}})=\exp (-\frac{\parallel {P}_{{x}_{i}}-{P}_{{t}_{j}}{\parallel }_{2}^{2}}{{h}^{2}})$$

where $h=\,{\rm{\min }}\,\parallel {P}_{{x}_{i}}-{P}_{{t}_{j}}{\parallel }_{2}^{2}+\varepsilon$ and $\varepsilon \to 0$. The pathological status *p*_*t*_ is set to −1 for patches extracted from AD patient and to 1 for patches extracted from CN subject. Therefore, the PBG method provides a score representing an estimation of the alterations caused by AD at each voxel. Consequently, cerebral tissues strongly altered by AD have grading values close to −1 contrary to healthy one with scores close to 1.

### Multimodal patch-based grading fusion

The patch-based method presented in the previous section was designed to capture structural alterations in T1w MRI. Recently, we proposed the extension this method to DTI modality in order to detect microstructural modifications^65^. We showed the efficiency of MD grading in improving the classification of the early stages of AD.

**Figure 2:**
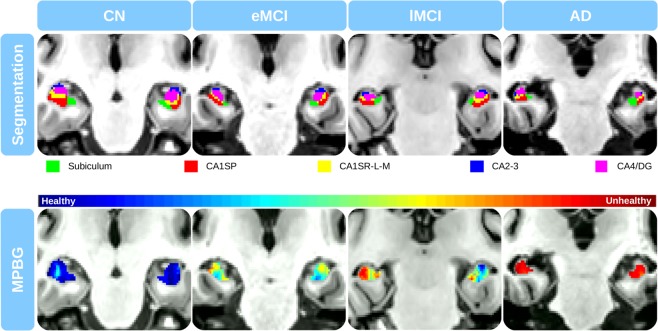
The presented results have been obtained using MRI from patients suffering from different severities of cognitive impairments. From top to bottom slices on the coronal plane of the segmentation maps, and the fusion of T1w and MD patch-based grading (*i*.*e*., MPBG: Multimodal Patch-Based Grading) with the proposed multimodal patch-based grading method. The blue and red colors represent healthy and altered tissues, respectively. To avoid bias due to overlap between training and testing datasets, the library has been constructed within a leave-one-out procedure.

In this study, we propose a new framework to perform multimodal patch-based grading (MPBG). To this end, we developed an adaptive fusion of grading maps derived from different modalities (see the example of grading maps on Fig. 2). As shown in the following, this fusion provides more robust and accurate biomarkers compared to monomodal PBG biomarkers.

As in the previous section, a training library of CN and AD subjects is built for each modality. Next, at each voxel within the ROI of the considered subject and for each modality, a set *K* of most similar patches is extracted. This step provides one set *K* of patches per modality $m\in M$, where *M* corresponds to the set of the different modalities provided. Nevertheless, at each voxel, the quality of the grading estimation is not the same for all the modalities. Therefore, the degree of confidence is estimated with the function *α* defined as:

3 $${\alpha }_{{x}_{i,m}}=\sum _{{t}_{j}\in {K}_{{x}_{i,m}}}\,w({P}_{{x}_{i,m}},{P}_{{t}_{j,m}})$$

that reflects the confidence of the grading value ${g}_{{x}_{i}}$ for the modality *m* at the voxel *x*_*i*_. This confidence measure is derived from multi-feature fusion^86^. Thus, each modality provides a weak classifier at each voxel that is weighted with its degree of confidence ${\alpha }_{{x}_{i,m}}$. The multimodal grading denoted ${g}_{{x}_{i}}$, is given by:

4 $${g}_{{x}_{i}}=\frac{{\sum }_{m\in M}\,{\alpha }_{{x}_{i,m}}{g}_{{x}_{i,m}}}{{\sum }_{m\in M}\,{\alpha }_{{x}_{i,m}}}.$$

In other words, the weights *w* and ${K}_{{x}_{i,m}}$ are estimated independently for each modality and combined afterward. Therefore, the proposed combination framework is spatially adaptive and takes advantage of the a local degree of confidence ${\alpha }_{{x}_{i,m}}$ for each modality *m*. When the matches found for a modality in the training library is composed of good candidates (*i*.*e*., patches very similar to the patch from the subject under study), our confidence ${\alpha }_{{x}_{i,m}}$ in the grading estimation for this modality is high. In the end, this modality will have a high weight in the mixing procedure described in (4).

### Features estimation

**Figure 3:**
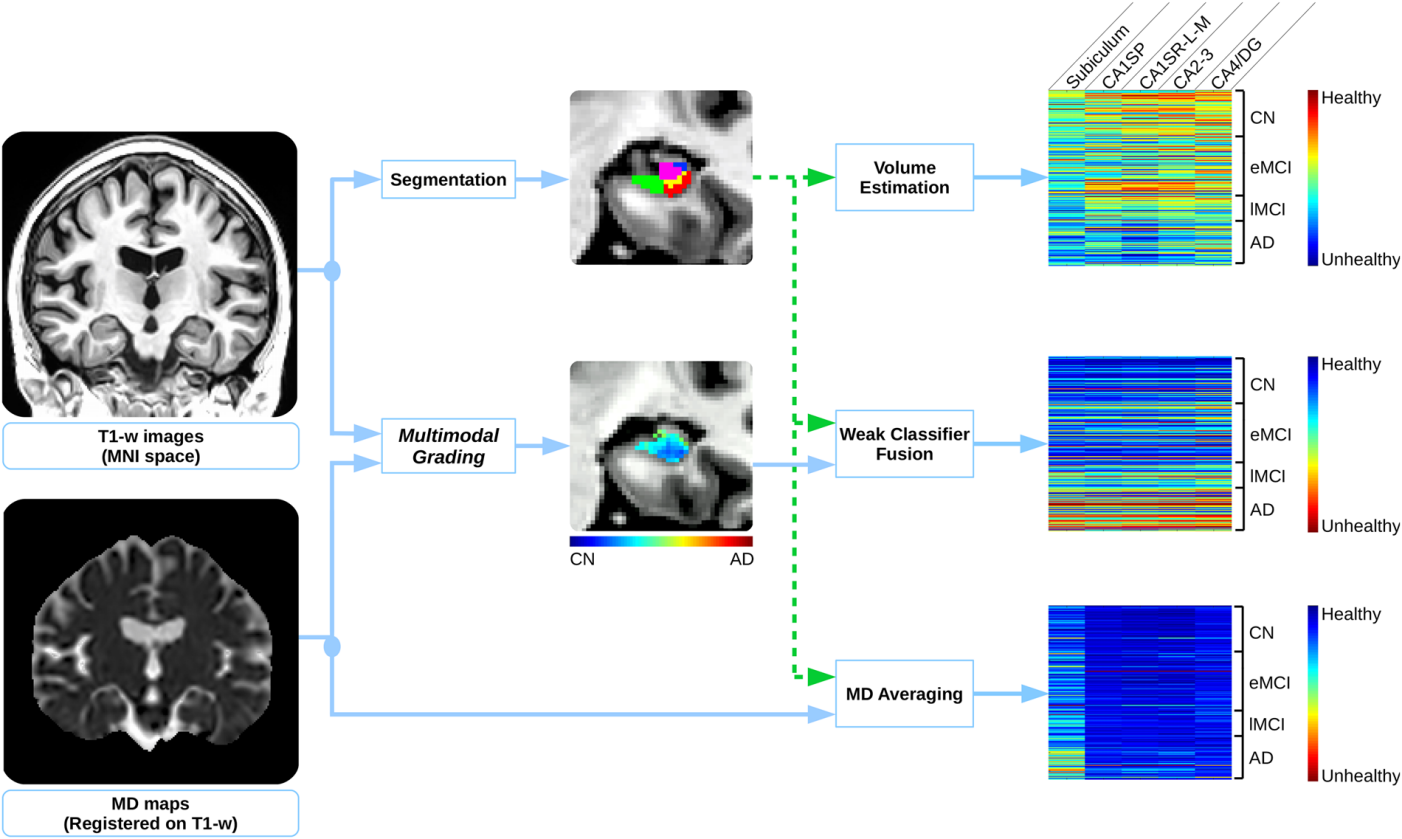
Proposed multimodal patch-based grading framework. At left, the input data: T1w images and MD maps into the MNI space. Data represented in this figure belongs to a CN subject. At the middle: a coronal view of hippocampal subfields segmentation on T1w, and the corresponding coronal view of a multimodal patch-based grading (MPBG) map estimated on T1w and MD. At right, the considered subfield biomarkers for all subjects under study. From top to bottom, the features are the volumes, the MPBG values, and the average of MD.

Features were estimated in each hippocampal subfield and over the whole hippocampus as the union of all hippocampal subfields masks. To reduce the inter-individual variability, all volumes are normalized by the total intra-cranial volume^87^. Afterward, we aggregate weak local classifiers of the grading map into a single feature for each considered structure (*i*.*e*., hippocampal subfields, and whole hippocampus) by averaging them. Then, patch-based grading features are computed by an unweighted vote of the weak classifiers using the segmentation masks (see Fig. 3). Finally, to prevent the bias introduced as the structural alterations due to aging, all the features (*i*.*e*., volume, mean of MD and MPBG) are age corrected with a linear regression based on the CN group^88^.

### Implementation

We use the OPAL method to find the most similar patches in the training library^89^. OPAL is a fast approximate nearest neighbor patch search technique. This method processes each modality in about 4 seconds on a standard computer. A leave-one-out procedure was followed to construct the training library. Hence, for each test subject, a different training library is built. Consequently, the training library *T* is composed of 37 images from CN subjects and 37 images from AD subjects, for a total of 76 images. The number of patches extracted from both training libraries is *K* = 160 (*i*.*e*., 80 from CN subjects and 80 from AD patients) and the patch size is 5 × 5 × 5 voxels.

Furthermore, as done in our PBG DTI study^65^, we used zero normalized sum of squared differences for T1w to compute the L2 norm (see Eq. (2)). On the other hand, d-MRI is a quantitative imaging technique. Therefore, a straight sum of squared differences is used for MD in Eq. (2) in order to preserve the quantitative information.

### Validation

To evaluate the efficiency of each considered biomarker in detection of AD alterations, the CN group is compared to the group of AD patients. In addition, to discriminate the impairment severity of MCI group, eMCI versus lMCI classification is conducted. The classification step is performed with linear discriminant analysis (LDA) within a repeated stratified 5-fold cross-validation with 200 iterations. Mean area under the curve (AUC) and mean accuracy (ACC) are computed to compare performance for each biomarker over the 200 iterations.

### Statistical analyses

Statistical tests were conducted with an analysis of variances (ANOVA) procedure to determine the significance of biomarkers changes, related to the alterations caused by AD. The results of these tests have been corrected for multiple comparisons with Bonferroni’s method. Significant changes have been tested within six comparisons (*i*.*e*., CN-AD, CN-eMCI, CN-lMCI, eMCI-lMCI, eMCI-AD, and lMCI-AD). These comparisons have been achieved into each region of the hippocampus and with the three considered biomarkers (*i*.*e*., the volume, the average of MD, and our newly proposed MPBG). Finally, for each iteration of our stratified 5-fold cross-validation, we estimated the confidence interval of AUC using bootstrap iterated for 100 iterations^90^. Then an average of the minimum and maximum bounds are computed. The results presented in this paper show the average confidence interval based on these average bounds.

## Results

In this section, the results are presented in three parts. In the first part, we compare the different approaches applied within the entire hippocampus structure to evaluate the performance of our new MPBG compared to usual biomarkers such as volume and average MD. In the second part, we compare the accuracy of each considered biomarker within hippocampal subfields in order to investigate the potential of hippocampal subfield analysis to improve the result of AD detection and prediction. Finally, we compare the results of our proposed multimodal biomarker with state-of-the-art methods based on d-MRI to show the competitive performance of our approach.

### Whole hippocampus

Results of the comparisons over the whole hippocampus are presented in Table 2. In this experiment, we compared the results of volume, mean of MD and PBG applied with both modality and MPBG over the whole hippocampus.

**Table 2:** Mean AUC of the different features estimated over the whole hippocampal structure.

Method	CN vs. AD	eMCI vs. lMCI
Volume	86.6	59.4
MD	80.6	55.6
T1w PBG	**92.6**	67.5
MD PBG	89.2	**69.5**
MPBG	92.1	**69.5**

In bold font, the best result for each specific comparison. All results are expressed in percent. The results presented in this table show that patch-based grading method (PBG) applied on T1w obtains better result compared to PBG applied on MD for CN vs. AD classification while MD PBG obtains best results for eMCI vs. lMCI classification. Finally, multimodal patch-based grading (MPBG) applied on T1w and MD obtains similar results compared to the best performances of both modalities.

First, the hippocampus volume and its average of MD were compared. For CN versus AD classification, the volume obtains 86.6% of AUC, and the average of MD obtains 80.6%. For eMCI versus lMCI classification, the volume and the average of MD obtain 59.4% and 55.6% of AUC, respectively. The experiments demonstrate that the volume of the hippocampus results in better classification performances than the average of MD for all comparison, especially for CN versus AD. Second, PBG biomarkers applied with T1w and MD were compared. The results showed that T1w PBG provides better results than MD PBG with 92.6% of AUC for CN versus AD classification. However, for eMCI versus lMCI classification MD grading provides the best results with 69.5% of AUC. MPBG methods combining both modalities performed similarly to the best results for CN versus AD and eMCI versus lMCI with 92.1% and 69.5% of AUC, respectively. Finally, the proposed MPBG biomarker provides results similar to the best modalities for all considered comparisons. MPBG improves CN versus AD comparison result by 5.5% of AUC and by over 10% of AUC for eMCI versus lMCI comparison. Thus, MBPG biomarker has a good capability to capture modifications caused by AD at different stages of severity (see Fig. 2).

### Hippocampal subfields

**Figure 4:**
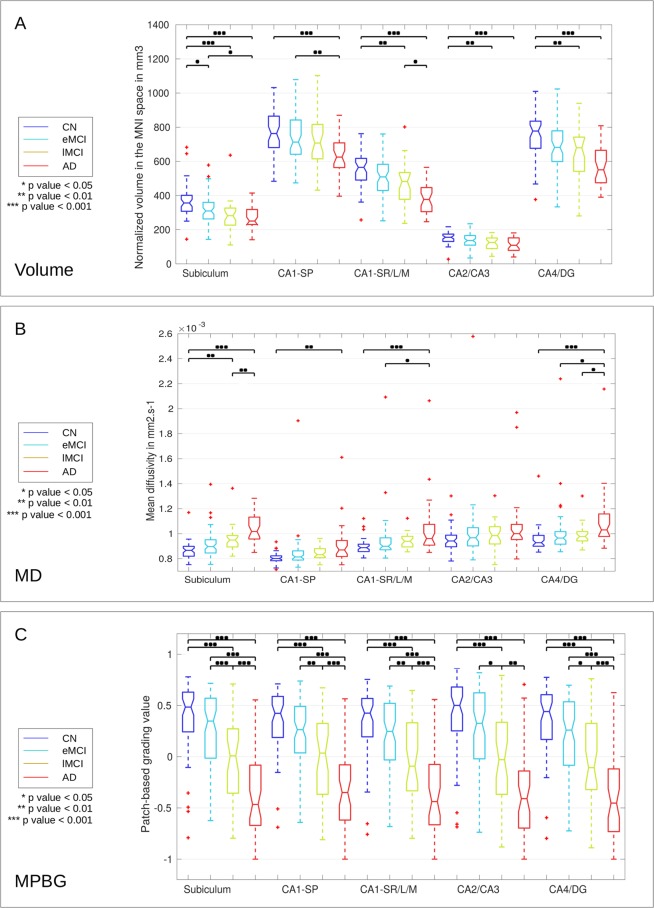
Distribution of the volume (**A**), MD (**B**), and MPBG (**C**) for the different considered groups. The normalized volumes are provided in *mm*^3^ in the MNI space for each subfield, MD is the mean of MD values into each subfield in *mm*^2^.*s*^−1^, and MPBG is the mean patch-based grading values into each subfield. Blue, cyan, orange, and red colors represent CN, eMCI, lMCI, and AD subjects, respectively. Statistical tests have been performed with ANOVA procedure and corrected for multiple comparisons with the Bonferroni’s method. The p-values inferior to 0.05, 0.01, and 0.001 are represented with *, **, and ***, respectively.

Figure 4 shows the distribution of volumes (A), the average of MD (B), and the MPBG (C) for each hippocampal subfield at different AD stages. For each comparison, a p-value was estimated with a multi-comparison test^91^. We can note that for all hippocampal subfields, alterations caused by the disease are related to volume and MPBG decrease with MD increase. The subiculum subfield presents the most significant differences for CN versus lMCI using volume and MD, for AD versus lMCI using MD, and for eMCI versus lMCI using MPBG. Indeed, it is the only subfield providing a p-value inferior to 0.05 for the comparison of CN versus eMCI using volume, a p-value inferior to 0.01 for lMCI versus AD using MD and a p-value inferior to 0.001 to eMCI versus lMCI using MPBG, which are the most challenging comparisons. The distribution of MPBG shows better discrimination between each group for all hippocampal subfields. Indeed, MPBG applied within CA1SP, and CA1SR-L-M provides p-values inferior to 0.01 for eMCI versus lMCI. Moreover, MPBG applied within the subiculum provides p-value inferior to 0.001 for the same comparison. Thus, MPBG enables AD detection using each subfield with an advantage for subiculum for the comparison of eMCI versus lMCI.

**Figure 5:**
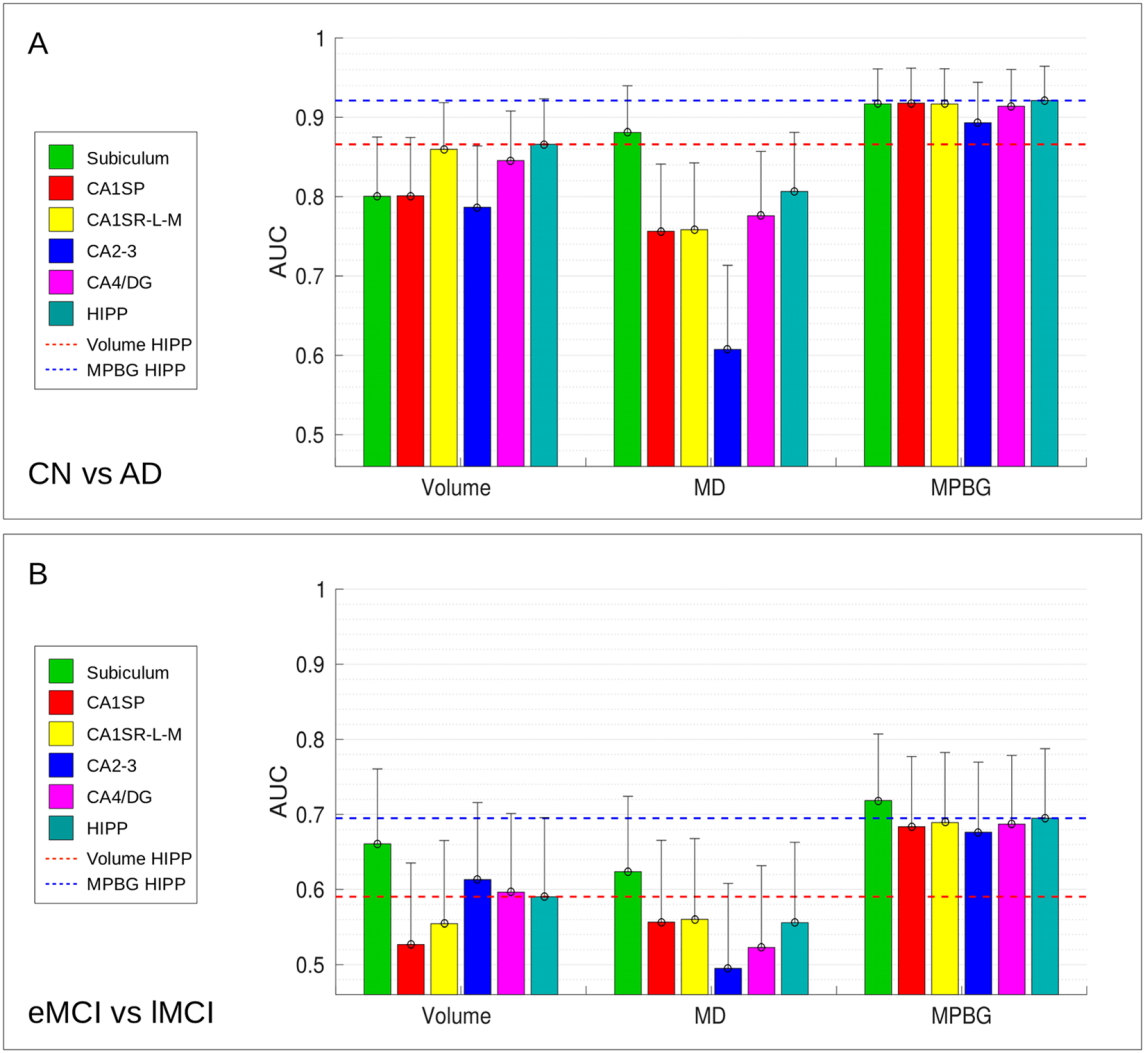
AUC computed for CN versus AD (**A**), eMCI versus lMCI (**B**) comparisons with the different considered biomarkers in each hippocampal area. Results of subfields are grouped by features (*i*.*e*., the volume, the average of MD and the MPBG). Upper bounds of the confidence interval are represented with vertical bars. Whole hippocampus volume biomarker provides the best results with a mean AUC of 86.6% for CN versus AD comparison, followed by the CA1S-R-L-M volume that obtains a mean AUC of 86%. Subiculum volume provides the best results for eMCI versus lMCI with a mean AUC of 66.1%. The average of MD for subiculum obtains the best results for CN versus AD and eMCI versus lMCI with a mean AUC of 88.1% and 62.4%, respectively. Whole hippocampus MPBG obtains the best results for CN versus AD with a mean AUC of 92.1%. Subiculum MPBG obtains the best results for eMCI versus lMCI comparison with a mean AUC of 71.8%. This comparison shows that subiculum is the only biomarker providing better results than the whole hippocampus. This figure presents mean AUC and the mean confidence intervals that have been computed for each iteration of the stratified 5-fold cross-validation procedure carried out in our experiments.

To estimate the efficiency of the considered biomarkers for AD detection, we also performed a classification experiment. Figure 5 shows the results of two comparisons, CN versus AD (part noted A in the figure) and eMCI versus lMCI (part noted B). First, for AD diagnosis (*i*.*e*., CN versus AD classification), the subfield providing the most discriminant volume is the CA1S-R-L-M with an AUC of 86.0%. Moreover, the most discriminating MD biomarker is given by the subiculum with an AUC of 88.1%. For this comparison, the MD of subiculum is the only biomarker performing better results than the whole hippocampus. The CA1SP provides the best results using MPBG feature with an AUC of 92.1%, followed by the CA1S-R-L-M and the subiculum.

Second, for eMCI versus lMCI classification, the subiculum provides the best results for each considered feature. Indeed, the subiculum obtained an AUC of 66.1% for the volume, 62.4% for the average of MD, and 71.8% for MPBG. Moreover, the subiculum also provided better results than the whole hippocampus for each considered method. Thus, the experiments conducted with three different biomarkers showed that the use of hippocampal subfields, especially the subiculum, results in better AD prediction than the whole hippocampal analysis.

### Comparison with state-of-the-art methods

Direct comparison with other monomodal methods applied on ADNI1 is difficult since group definition (stable MCI and progressive MCI) are different. However, as recently shown, T1w PBG provides state-of-the-art performance on ADNI1 dataset, even compared to deep learning methods^92^. Consequently, the results presented in this paper with T1w PBG on ADNI2 can reasonably be considered competitive and can be used as a reference.

Consequently, to evaluate the performance of the proposed MPBG, we compared it with state-of-the-art multimodal methods using d-MRI. To this end, we used the ACC values published by the authors. Table 3 shows the comparison of our proposed biomarkers within the hippocampal area providing the best results (*i*.*e*. the whole hippocampus and the subiculum) with the state-of-the-art methods using similar dataset based on ADNI-2. We compared these biomarkers with a method using features based on tractography^93^, two different methods based on connectivity networks of the different brain structures^60,94,95^, and a voxel-based method that analyzes alterations of white matter^96^. The results of the comparison show that MPBG over the whole hippocampus obtains the best score for AD versus CN with 88.1% of accuracy while the best result is achieved by a voxel-based method with a feature selection^96^ that obtained 87.0% on similar ADNI2 dataset. For the best of our knowledge, the two works providing eMCI versus lMCI comparison^60,94^ using s-MRI and d-MRI from a similar ADNI2 dataset are based on a connectivity network and obtained 63.4% and 65.0%, respectively. These comparisons demonstrate the relevance of MPBG biomarkers for AD detection and prediction. Indeed, our method provides similar results than the best methods with similar dataset for CN versus AD classification and provides the best results for eMCI versus lMCI classification. Moreover, the proposed MPBG method based on the subiculum improves the performance for eMCI versus lMCI classification with an accuracy of 70.8%, that increases by 2% the accuracy based the whole hippocampus and over 6% compared to a connectivity network-based method.

**Table 3:** Comparison of our proposed MPBG biomarkers with state-of-the-art methods based on s-MRI and d-MRI using a similar ADNI2 dataset.

Method	Subjects				Feature	Classifier	Classification ACC	
	CN	eMCI	lMCI	AD			CN/AD	eMCI/lMCI
Nir *et al*.^93^	44	74	39	23	Tractography	SVM	84.9%	n/a
Prasad *et al*.^60^	50	74	38	38	Connectivity network	SVM	78.2%	63.4%
Zhan *et al*.^94^	n/a	73	39	n/a	Connectivity network	SLG	n/a	65.0%
Maggipinto *et al*.^96^	50	22	18	50	Voxel-based	RF	87.0%	n/a
La Rocca *et al*.^95^	52	85	38	47	Connectivity network	RF	83.0%	n/a
MPBG hippocampus	62	65	34	38	Patch-based	LDA	**88.1%**	68.8%
MPBG Subiculum	62	65	34	38	Patch-based	LDA	86.5%	**70.8%**

All results are expressed in percentage of accuracy.

LDA = Linear Discriminant Analysis,

SLG = Sparse Logistic Regression,

SVM = Support Vector Machine,

RF = Random Forest.

### Relationship with cognitive scores

To investigate relationships between cognitive scores and MPBG values, we performed a generalized linear analysis with the following model: *MPBG* = *β*_0_ + *β*_1_.*ages* + *β*_2_.*sex* + *β*_3_.*MMSE* + *β*_4_.*RAVLT* + *β*_5_.*FAQ* + *β*_6_.*CDRSB* + *β*_7_.*ADAS*11 + *β*_8_.*ADAS*13. We found significant relationship of hippocampal MPBG with sex (p < 0.01), MMSE (p < 0.05) and ADAS 13 (p < 0.01). This correlation with MMSE and ADAS scores is valid for all subfields of the hippocampus. We found no specific model for a given subfield, all presented a similar pattern. These results are in line with relationships obtained between hippocampus subfields volumes and MMSE and ADAS^97^.

## Discussion

In this work, multimodal analysis of the hippocampal subfields alterations caused by AD is proposed. First, the structural and microstructural alterations were captured from two MRI modalities with different methods. Then, the use of volume, MD, and the proposed MPBG methods were investigated to achieve this analysis. In this section, the efficiency of these different methods applied to the whole hippocampus, and each hippocampal subfield are discussed.

### Whole hippocampus biomarkers

We first compared the performance of different methods applied to the whole hippocampus (see Table 2). The experiments showed that volume and average of MD of the hippocampus do not provide the most discriminating biomarkers to detect early stages of AD. Indeed, the proposed MPBG method obtains better results compared to the volume and the average of MD. However, for CN vs. AD, our MPBG method obtained lower results than T1w PBG when applied to the hippocampus. Therefore, the substantial structural differences between these two populations seem to be better captured using T1w modality. This probably comes from the better native resolution of this modality. On the other hand, for eMCI vs. lMCI, MPBG and MD PBG obtained the best result. Therefore, the subtle alterations between both populations seem to be better captured using DTI modality. This may come from the capability of this modality to measure microstructural modifications. Finally, when applied on the whole hippocampus, our MPBG demonstrates state-of-the-art performances for AD detection and prediction hippocampus compared to recent methods (see Table 3).

These results emphasize the relevance of using more accurate biomarker, such as MPBG, to study the effectiveness of hippocampal subfields for AD detection and prediction.

### Hippocampal subfield biomarkers

The main contribution of this study is the multimodal analysis of hippocampal subfields. Indeed, most of the proposed biomarkers based on the hippocampus focus only on the whole structure or study alterations of hippocampal subfields with methods that do not provide sensitive biomarkers to detect early modification caused by AD. The lack of work studying alterations of hippocampal subfields with advanced biomarkers could be explained by the fact that automatic segmentation of the hippocampal subfields is a complex task due to subtle borders dividing each area.

In this work, we compared the efficiency of diffusion MRI and multimodal patch-based biomarkers for AD detection and prediction over the hippocampal subfields. Comparisons based on MD, volume and multimodal patch-based biomarkers showed that the subiculum is the most discriminating structure in the earliest stage of AD providing the best results for AD prediction (see Figs 4 and 5). However, whole hippocampus structure, followed by CA1SR-L-M, obtains best results for AD detection.

These results are in accordance with literature studies based on animal model and *in vivo* imaging combining volume and MD demonstrating that the subiculum is the earliest hippocampal region affected by AD^49,50^. Moreover, postmortem studies showed that hippocampal degeneration in the early stages of AD is not uniform. After the apparition of alterations in the EC, the pathology spreads to the subiculum, CA1, CA2-3 and finally the CA4 and DG subfields^43,44,49,98^. It is interesting to note that the results of our experiments using volume-based biomarkers are also coherent with the previous *in*-*vivo* imaging studies that analyzed the atrophy of each hippocampal subfield at the advanced stage of AD. These studies showed that CA1 is the subfield impacted with the most severe atrophy^45,46,99,100^. Furthermore, studies using the ultra-high field at 7T, enabling CA1 layers discrimination showed that CA1SR-L-M are the subfields showing the greatest atrophy at advanced stages of AD^47,48^.

### Comparison with state-of-the-art methods

In the past years, a large number of studies dedicated to automatic detection of Alzheimer’s disease have been proposed^53,69,93,101^. For a fair comparison, we consider only methods based on similar modalities and validated on the same ADNI2 dataset. Direct comparison with other monomodal methods applied on ADNI1 is difficult because group definition and pathological status definition are different. However, we can observe that the results obtained by the proposed method are in line with recently published results for AD vs. CN^102^.

### Strengths and limitations

The major strength of our work comes from studying the effectiveness of using multimodal hippocampal subfields alterations for AD classification with a novel multi-modal patch-based grading framework. Nonetheless, we acknowledge that our multi-modal framework is not without potential limitations. The main limitation is the large voxel size of DWI in native space that is prone to PVE by merging signal from CSF with the signal from brain tissues. This results in an increase of MD coefficients, especially for structures with severe atrophies. However, to limit this aspect, we corrected the PVE^83^. Indeed, it has been shown that the use of up-sampling methods over individual DWI direction enables reduction of the PVE effect. Nevertheless, this study does not aim to provide an interpretation of DTI parameters modification, but to study the effectiveness of the use of hippocampal subfields for AD classification with multimodal patch-based grading method. Finally, although our method extracts patches independently from both s-MRI and d-MRI modalities to estimate grading maps from both modalities, the fusion of the two grading maps requires accurate alignment of images from each modality. Consequently, the correction of EPI distortions is crucial in ensuring that each voxel corresponds to the location.

## Conclusion

In this paper, we analyzed hippocampal subfield alterations with a multimodal framework based on structural and diffusion MRI. In addition, to study tenuous modifications occurring in each hippocampal subfield, we developed a new multimodal patch-based framework using T1w and DTI. Our novel MPBG method was compared to the volume and the average of MD over the whole hippocampus. This comparison demonstrated that our MPBG method improves performances for AD detection and prediction. Also, a comparison with state-of-the-art diffusion-based methods showed the competitive performance of MPBG biomarkers. Finally, volume, average MD and MBPG methods were used to analyze hippocampal subfields. Although CA1 is the subfields with the greater atrophy in the late stage of AD, the experiments demonstrated that the whole hippocampus provides the best biomarker for AD detection while the subiculum provides the best biomarker for AD prediction.

## Data Availability

The datasets generated during and/or analyzed during the current study are available from the corresponding author on reasonable request.
